# Supplementary material for: Are carbon emissions trading and green financial instruments synergistic? -Comprehensive quantitative research based on content analysis
Source: PLoS One. 2024 Mar 7;19(3):e0298601. doi: 10.1371/journal.pone.0298601 (PMC10919601; doi:10.1371/journal.pone.0298601)
Supplement: S1 Data — (ZIP) [file pone.0298601.s001.zip › Data/地级市Z_F工作报告.zip!/║ú─╧/║ú┐┌╩╨/└┤╫╘╣1⁄2╓┌║┼í╛┬φ┐╦╩2╛▌═°í┐_2008.docx]

各位代表：

　　现在，我代表市人民政府向大会作工作报告，请予审议，并请各位政协委员和列席人员提出意见。

　　一、2007年的主要工作

　　2007年，在省委、省政府和市委的正确领导下，我们以科学发展观统领全局，深入实施工业化、城市化、现代物流业、休闲娱乐业“四轮驱动”战略，创新体制机制，提升城市品位，推进新农村建设，切实解决民生问题，积极促进社会和谐，经济社会继续保持又好又快的发展态势。全市生产总值达396.35亿元，增长12.6%;财政总收入达到82.5亿元，增长17.7%，地方财政收入达31.29亿元，增长19%;全社会固定资产投资182.77亿元，增长15.5%;城镇居民人均可支配收入12289元，增长14.7%，农民人均纯收入4582元，增长10.3%，城乡居民收入增幅创10年来新高。较好完成了市十四届人大一次会议确定的目标任务，实现了本届政府的良好开局。

　　(一)经济结构进一步优化，优势产业加快发展

　　一年来，我们坚持稳定优化一产，调整提升二产，加快发展三产，结构调整和产业升级有新进展。

　　特色农业发展加快。完成农业增加值29.5亿元，增长8%。农业结构进一步优化，标准化生产基地和出口示范区建设初具规模，通过省级认定的无公害农产品基地48个，新建田头冷库3个，荔枝、香蕉、罗非鱼等农副产品出口、出岛大幅增加，文昌鸡活鸡实现直接供港。优势农产品对农业经济增长的贡献率达到88%，畜牧业产值占农业总产值的比重达40%。

　　工业规模进一步壮大。完成工业增加值86.41亿元，增长12.7%。海马二期基础设施建设基本完成，药谷二期、狮子岭飞地工业园建设加快推进，新增入园企业21家。全市规模以上工业企业已达192家，产值超亿元的企业达55家，规模以上工业对经济增长的贡献率进一步提高。

　　服务业发展较快。完成第三产业增加值255.75亿元，增长13.6%。发起建立“琼北湛江区域旅游合作组织”，搭建了区域旅游合作新平台;组织“十万人自驾游海南”、“中国热气球飞越琼州海峡挑战赛”、“全国速度轮滑公开赛”等一系列大型活动;加大对航空公司和旅行社的奖励，接待国内外过夜旅游者585.65万人次，增长9.2%;旅游总收入55.39亿元，增长8.1%。精心组织海口地产开发论坛暨政府土地储备项目推介会，房地产业健康发展，全年房屋销售面积172.99万平方米，增长22.1%。会展经济迅猛发展，专业会议和大型会展达6398场次。消费品市场持续走旺，居民消费结构不断升级，社会消费品零售总额达到189.38亿元，增长17.4%。金融保险、信息服务、物流、软件等产业快速发展。

　　(二)重点改革取得突破，对外开放迈上新台阶

　　一年来，我们积极推进和深化重点领域改革，提高对外开放水平，发展活力明显增强。

　　体制创新迈出新步伐。行政管理体制改革取得重大突破，认真落实市委《关于创新市区管理体制实施强区扩权的决定》，首批54项行政权项的下放和衔接工作正在有序推进。投融资体制改革取得重大成绩，以9.3亿元成功出让49%的水务集团国有股权，溢价202%，成立海口威立雅水务有限公司。整合全市城建资产，成立城建集团。市政道路清扫保洁以及水域垃圾管理实现环卫作业社会化管理。

　　对外开放水平有新提高。利用外资稳步增长，实际利用外商直接投资5.04亿美元，增长12.8%。进出口势头良好，外贸进出口总额19.34亿美元，增长44.1%。保税区区位调整报批工作有重大进展。积极参与第四届“珠洽会”、泛珠省会城市市长论坛等经贸洽谈活动，区域合作卓有成效。新增国际友城2个，成为世界城市和地方政府联合组织新成员。

　　(三)基础设施建设明显加快，城市环境进一步改善

　　一年来，我们加强重点项目建设，建成一批对今后发展具有重大意义的基础设施项目，城市品质不断提升。

　　重大基础设施建设加快推进。完成政府投资45亿元，增长50%。和平桥、人民桥、海南广场周边路网、国兴-海府立交等投入使用;绕城高速、长秀片区路网、海甸岛环岛路、海口港二期等项目建设进展顺利;长流起步区路网、东环铁路海口段、新海大桥的建设已启动，马村港一期扩建工程开工建设。

　　旧城改造和新片区开发取得新突破。按照“政府主导”和“让利于民”的原则，配套完善多种鼓励措施，玉沙村改造取得重大进展，为旧城改造积累了经验。

　　完成海甸溪北岸、长堤路改造和五条历史文化街保护的前期工作。大英山、美丽沙、西海岸、新埠岛开发建设全面启动。

　　城市环境明显改善。三年环境综合整治取得阶段性成果，去年拆除违法建筑33.5万平方米，违法建筑行为得到进一步遏制。对和平大道、龙昆北路等一批主干道实施街景立面改造，“一园两湖”整治进展顺利。全面启动污染源普查，继续抓好市中心区污水截留并网工程，完成颜春岭垃圾填埋场垃圾渗滤液处理工艺改造。新增各类绿地240多公顷，建成区绿地率和绿化覆盖率分别达到35.9%、40%。顺利通过国家环保模范城复查现场验收，成为全国第一个通过新标准复检的城市。

　　(四)统筹城乡发展，新农村建设呈现新局面

　　一年来，我们加快城乡经济社会一体化进程，加大投入，着力改善农业基础条件，新农村建设扎实推进。

　　支农惠农力度进一步加大。农林水利支出达2.05亿元，增长31%;对农业直接补贴2536.64万元，增长128%。农业科技110覆盖所有乡镇，23个镇都有电子农务服务站。农民专业合作社发展到97家。培训农民3万人次，转移农村富余劳动力1.35万人，打工经济进一步活跃。

　　农村生产生活条件明显改善。投入2400万元，完成一批田洋整治和水库的除险加固。投入近3000万元，进一步解决羊山地区缺水问题。投入1600多万元，建成防洪楼15幢。完成200多公里的乡村公路建设，农村自来水覆盖率达到93%以上，兴建12个大中型沼气池。公路绿化360公里，造林4万多亩。新增文明生态村170个，总数达982个，占自然村总数的46%，农村面貌发生可喜变化。

　　(五)更加关注民生，和谐社会建设稳步推进

　　一年来，我们始终把解决民生问题作为“一号工程”来抓，财政投入社会事业资金达10.86亿元，增长27.6%，办成一批事关群众切身利益的实事。

　　人民群众得到更多实惠。安排7.8亿元，全力推进27项为民办实事事项，除2项需跨年度完成外，其它全部完成。安排就业和培训费达4226万元，新增就业岗位3.2万个。建立零就业家庭援助长效机制，开发公益性岗位1200多个，第一批确认的零就业家庭全部得到安置。建立和健全城乡居民最低生活保障制度和生活困难群众的救济机制，累计发放低保金4003万元，城乡低保实现动态管理下的应保尽保;针对物价上涨，提高低保对象物价补贴标准，惠及全市低保对象近5.2万人。动工兴建1014套廉租房，向城市低收入困难家庭发放租赁补贴。

　　社会事业发展成绩突出。深入实施“科教兴市”战略，专利申请量、专利授权量分别增长 49%、26%，新认定9家省级高新技术企业，荣获“全国科技进步先进市”称号。教育布局进一步优化，新增优质学位6000多个，加快推进旅职校和四个区职业教育中心建设，教育质量和水平有了新提高。加强疾病预防控制，公共卫生体系进一步健全，55万农民参加农村合作医疗，参合率达98%以上，获得全省新型农村合作医疗考核一等奖;完成城镇居民基本医疗保险试点工作，35万多人参保，参保率达95%。文化事业发展繁荣，组织开展38场精品演出、群众性文化活动3000多场次，《大明王朝1566》获得中国电视剧最高奖“飞天奖”，陈素珍获中国戏剧最高奖“梅花奖”。群众体育、竞技体育取得新成绩，在第六届全国城市运动会上获4金3铜的历史最佳成绩，在法国举行的“城市之间”国际版上获亚军。

　　加强人口与计划生育工作，保持低生育水平。创新双拥共建新模式，实现全国双拥模范城“六连冠”。妇女儿童、老年人、残疾人权益得到更好的保障，统计、民族、宗教、社会科学、外事侨务、防震减灾、地方志、档案、科普等工作都取得新成绩。

　　(六)政府自身建设得到加强，民主法制建设取得新成绩

　　一年来，我们坚持为民、务实、清廉，政府科学决策、民主执政、依法行政的能力不断增强。

　　民主法制进一步加强。自觉接受人大及其常委会监督、政协民主监督、社会舆论及人民群众监督，加强与各民主党派、工商联和无党派人士的沟通联系。完善市长接待日制度，开通“椰城纠风热线”。进一步完善社会公示与社会听证制度和政府新闻发言人制度。全年共办理人大代表议案、建议204件、政协委员提案303件。全面落实“五五”普法教育，社区居民自治、村民自治及基层民主政治建设稳步推进。圆满完成村级组织换届选举工作。

　　行政效能得到提升。电子政务建设成效明显，初步实现“一网式”办公和“一站式”服务。优化政府管理流程，政府常务会议议定事项办结率达98%。加大政府目标管理和督查力度，严格执行行政问责制。实施重点项目推进联席会议制度。加强工程招标、土地招拍挂、产权交易、政府采购等关键环节的监管。切实纠正损害群众利益的不正之风，廉政建设取得新成效。

　　社会保持稳定和谐。社会治安防控体系建设进一步加强，严厉打击各类违法犯罪活动，“两抢一盗”等影响群众安全的多发性侵财案件明显下降，群众对社会治安的满意率达94%，排在全国省会城市前列。畅通群众诉求渠道，信访秩序明显好转，群体事件下降13.4%。加强政府应急机制建设，应急预防和处置能力得到提高。深入开展产品质量和食品安全专项整治活动，全面完成国家和省确定的整治任务。健全安全生产责任制，挂牌整治各类安全隐患，安全生产形势总体平稳。

　　各位代表，2007年，是我市认真贯彻科学发展观，创新发展思路，实现又好又快发展的一年;是城乡基础建设投入最多，旧城改造和新区开发同步推进，城乡面貌变化最大的一年;是体制创新取得重大突破，重点改革顺利推进，发展活力显著增强的一年;是更加关注民生，切实改善居民生活，人民群众得到更多实惠的一年。我市在全面建设小康社会的道路上迈出了坚实的步伐。

　　这些成绩的取得，是在市委的正确领导下，在市人大和市政协的支持、参与、监督下，全市人民团结奋斗的结果。在这里，我谨代表市政府，向全市人民，向给予政府工作大力支持的人大代表和政协委员，向各民主党派、工商联和社会各界人士，向驻市人民解放军、武警部队官兵，向一切关心、支持海口发展的海内外朋友，表示衷心的感谢!

　　在看到成绩的同时，我们也认识到，当前我市经济社会发展中仍然存在不少问题和矛盾：结构性物价上涨比较明显，加重了低收入群体和特困家庭的生活困难;就业和再就业压力仍然较大，城乡居民收入相对偏低;能源短缺对经济发展的制约不断增加;社会管理还不够到位，影响稳定的因素依然存在;产业投资不足，社会投资不够活跃;政府作风建设等都存在一些问题。对此，我们必须高度重视，采取得力措施，认真加以解决。

　　二、2008年政府工作的总体要求和预期目标

　　今年，是全面贯彻落实党的十七大精神的第一年，是全面完成“十一五”规划承上启下的重要一年，我们迎来了改革开放30周年和建省办经济特区20周年，做好今年的工作，意义重大。我们必须主动顺应宏观调控形势，坚持好字优先不动摇，坚持加快发展不动摇，坚持解放思想不动摇，坚持改革开放不动摇，发挥自身优势，创造条件，抢抓机遇，全力推动经济社会发展再上新台阶。

　　今年政府工作的总体要求是：深入贯彻党的十七大和省第五次党代会精神，以科学发展观统领经济社会发展全局，全面落实市第十一次党代会的各项工作部署。进一步调整优化产业结构，发展壮大优势产业;进一步推进重点改革，营造更具活力的体制机制;进一步争创生态环境新优势，提升城市品位;进一步改善民生，加快社会事业发展，努力构建和谐海口，推动经济社会又好又快发展。

　　根据总体要求，今年经济社会发展的主要预期目标是：全市生产总值增长12%左右;地方一般预算收入增长13%左右;全社会固定资产投资增长15%左右;城镇居民人均可支配收入、农民人均纯收入分别增长8%左右;城镇登记失业率控制在4%以内;居民消费价格总水平涨幅力争低于去年实际涨幅;人口自然增长率控制在8‰以内;每万元地区生产总值能耗下降2.5%。

　　三、积极实施“产业兴市”战略

　　按照“一产做优、二产做大、三产做强”的要求，统筹三次产业发展，以大项目带动大产业，以大产业壮大经济实力。

　　(一)着力发展现代农业，切实推进新农村建设

　　提升都市型农业发展水平。突出“无疫区、无公害”品牌，进一步调整农业产业结构，大力发展畜牧业、设施农业和农产品加工业，走高附加值的路子。加大对农业产业的扶持力度，安排专项资金，扶持发展设施农业，切实抓好优质瓜果菜、水产品、热带花卉、禽畜等规模化种植养殖出口示范基地建设。扶持壮大一批龙头加工运销企业，加快桂林洋农副产品出口加工区建设，启动新海港农产品物流配送中心项目，确保海南国际果蔬交易加工中心上半年投产。建立健全农业检测网络和农产品质量安全监管体系，确保农资产品和主要农产品抽检合格率达到 97%以上。全面深化电子农务体系建设，增强农业科技110、电子农务的服务功能，拓宽其覆盖范围。努力把海口建设成为全省农产品深加工中心，全省农产品物流中心，全省生态农业和科技农业示范基地，全省畜牧业、设施农业、农产品出口和标准化建设的大市，以及观光农业和三次产业联动的示范区。

　　切实增加农民收入。安排支农惠农专项资金7835万元，增长30.6%。组织实施新农村实用人才培训工程，继续加大外出务工农民职业技能培训力度，培训农民1.5万人次，提高农民致富能力，增加农民的生产性收入。采取多种措施发展打工经济，提高农民转移就业能力，农村劳动力转移就业1.2万人，增加农民的工资性收入。落实对农业生产直接补贴的各项政策，增加农民政策性补贴收入。

　　进一步完善农村生产生活条件。继续建设一批重大的农业基础设施工程。安排2000万元，基本解决5万农民安全饮水问题;安排1亿元，启动羊山地区生产用水工程;安排6000万元，完成268公里的农村公路建设，在全省率先实现行政村通硬化道路的目标;安排4000万元，加快中心镇建设，调整和优化镇域经济布局，把二、三产业引入中心镇。围绕发展农村经济、增加农民收入，创新文明生态村建设模式，安排500万元，新建文明生态村170个。

　　(二)大力发展新型工业，增强经济发展后劲

　　培育发展特色产业集群。挖掘和发挥现有企业的作用，依托现有的产业基础，延伸产业链，壮大以海马汽车为核心的汽车制造产业集群，以椰树、椰岛、亚太啤酒为核心的食品饮料及农副产品加工产业集群，以“药谷”为核心的医药产业集群，以盛之业、优美内衣为核心的化工化纤纺织产业集群，以海宇锡板、金盘电器、三星光缆为核心的机电制造产业集群，增强产业竞争力，形成一批优势更加明显的支柱产业。不断完善园区功能，投入1.7亿元，上半年完成药谷二期、狮子岭飞地工业园的基础设施建设;启动马村港保税物流园区和出口加工区的征地、拆迁和基础设施建设;积极筹划建设电子信息产业园。

　　发展高新技术产业。安排资金2125万元，继续培育高新技术企业，推进高新技术企业和技术创新示范企业快速成长，全年培育5家技术创新示范企业和5家重点实验室。建立风险投资机构和科技型企业投资担保体系，开展“一企一项”的技术改造和技术创新，重点扶持维瑅瑷生物研究院、先声药业等科研院所和高科技企业，研发一批具有自主知识产权的专利产品。

　　加大工业的扶持力度。建立市产业扶持基金，重点扶持优势产业和龙头企业，带动企业和社会投资，不断提高生产性投资的比重。加强工业经济运行调度监控，协调电、天然气等能源供给，积极发展太阳能产业，拓宽来源渠道，提高利用效能，确保重点企业、重大项目的需要。认真组织实施“优质服务年”系列活动，对工业企业建档立户，实行全程式主动服务，为企业发展创造良好的环境。

　　(三)加快发展现代服务业，提升城市服务功能

　　加快推进旅游业转型升级。完成《海口市旅游产业发展总体规划》的编制。加快建设羊山地区、美丽沙、新埠岛、海口湾、金沙湾等度假休闲旅游区，搭建户外有氧运动、主题公园、游客到访中心和自驾车营地等十大旅游平台，促进旅游基础设施上规模上档次。抓住建设旅游岛的有利时机，加快旅游业开放，对旅游要素实施国际化改造，推进旅游服务与国际接轨。上半年推出“印象海南岛”大型实景演出，继续组织定期定址的高品质活动，挖掘琼北湛江区域旅游资源，不断丰富旅游产品。整顿旅游市场，提高旅游管理服务水平，努力建设旅游目的地。加强旅游与房地产的融合，完善住房供应体系，优化商品房结构，扩大住房新的消费热点，促进房地产业健康发展。

　　扶持发展现代服务业。制定鼓励现代服务业发展的政策措施。积极推进现代物流业发展，建设以美兰机场为中心的空港经济区，以火车站为中心的物流区，以新港为中心的商业区，以马村港为中心的保税物流园区和出口加工区。壮大会展经济，建设国际会展中心，积极争办更多的全国性、区域性和国际性会展，扶持会展业做大做强。支持发展创意产业。大力发展电子商务、连锁经营等新的流通业态，扩大网络信息服务消费。扶持发展工程、管理、信息、咨询等中介服务业。逐步打造经营管理、商业贸易、金融保险、中介服务等四大现代服务中心，增强对全省的服务功能。

　　四、努力构建更具活力的体制机制

　　按照中央和省委、省政府的要求，全力推进重点改革，不断提高对外开放水平，切实增强发展的活力。

　　(一)推进重点领域改革，增强发展的体制动力

　　深入推进行政管理体制改革。加大强区扩权的实施力度，继续向区级下放管理权限，调整市区两级机构设置和人员编制，理顺市区财政体制，进一步增强区级发展动力。健全部门间协调配合机制，逐步形成高效运转的工作机制。深入开展事业单位机构改革和人事制度改革。

　　深入推进投融资体制改革。修订完善《政府投资项目管理暂行规定》，强化政府投资项目的组织、管理和监督。制定政府投资项目代建管理办法，营造代建制企业公平竞争的市场环境，吸引有实力的企业参与城市建设，逐步建立城镇基础设施项目投融资和运营管理的有效机制。在中央实施宏观调控政策的背景下，积极化解不利因素，努力吸引社会资本和民间资金，提高民营资本在总投资中的比重，保持必要的投资规模。

　　深入推进国有资产管理体制改革。积极探索国有资产运营的新模式，进一步整合国有资产，组建城投集团，深化城建集团运营机制改革，形成一批有竞争力的现代企业。支持水务集团整合琼山自来水公司，加快海峡航运、水务等集团公司的资本运营，努力培育新的上市公司，扩大企业融资渠道，放大国有资本。完善国有资产监管制度，规范国有资产的运营，确保国有资产保值增值。加快实施集体林权制度改革。支持配合农垦改革。

　　深入推进文化体制改革。认真落实我市《关于深化文化体制改革的决定》,按照政事分开、政企分开、管办分离的要求，理顺行业管理体制，推进文化事业单位的体制改革。拓宽融资渠道，实现投资主体多元化，政府重点投向满足市民公共文化产品与服务需求的文化设施领域，对公益性演出、高雅艺术的商业演出逐步实行补贴。

　　大力扶持发展中小企业和非公有制经济。着力落实我省《关于鼓励支持和引导个体私营等非公有制经济发展的若干规定》，进一步完善促进中小企业发展的政策措施，加快中小企业信用制度建设，扶持和鼓励一批高新技术企业到深圳中小板、创业板上市融资，着力解决中小企业发展的资金瓶颈。落实非公有制企业“收费检查卡”登记制度，切实维护投资者的合法权益。建立适合非公有制企业特点的信用征集体系、评价发布制度以及失信惩戒机制。对纳税大户和信用好、为社会作出突出贡献的非公有制企业和经营者实行表彰奖励，营造有利于非公有制经济发展的良好氛围。

　　(二)提高开放合作水平，构筑开放型经济新格局

　　大力开展招商引资。整合各类招商资源，整体策划招商工作，集中力量开展有针对性的专题招商活动，形成招商合力。突出招商重点，充分发挥企业主体作用和园区载体功能，着力提高先进制造业、现代服务业、现代农业和节能环保产业利用外资比重。创新招商引资方式，加大招商项目的策划、储备和推介力度，力争引进一批重大项目。

　　积极参与区域合作。加强基础设施、产业规划与周边县市的对接，积极构建以海口为中心的琼北综合经济区。强化琼北湛江区域旅游合作，拓宽合作领域，提高合作水平，积极打造琼州海峡两岸经济带。深化“9+2”泛珠三角区域在农业、旅游、贸易、科技等方面的合作，力争在区域合作中获得更多的发展机会。

　　五、着力推进温馨宜居城市建设

　　按照建设全国最精最美省会城市的总要求，坚持规划科学化、建设集约化、管理精细化，推动城市规划建设管理工作上新台阶。

　　(一)加强城市规划建设，提高城市品质

　　提高规划水平。统筹编制重大基础设施、生态环境、产业布局等规划。完成西海岸控制性规划、新港文化休闲中心区规划设计、海甸溪南岸街区修建性详细规划、历史文化名城保护规划的编制。切实强化规划的实施，依法加强执行监督。

　　完善城市综合功能。加快重大城市基础设施建设，全面完成绕城高速、滨江西路、长秀片区路网、省政府周边路网等项目建设，加快推进东环铁路海口段、丘海大道延长线、龙昆南路延长线、海甸岛环岛路等项目建设。做好重点项目建设前置性工作，统筹做好项目的征地、拆迁和安置房建设，确保重点项目建设顺利推进。全力推进西海岸起步区路网和污水管网建设，加快美丽沙、桂林洋高校区、金沙湾等新片区开发进度，做好滨江新城和江东组团开发的前期工作。玉沙村、海甸溪北岸、长堤路、五条历史文化街的改造要取得明显进展，继续实施主次干道的街景立面改造和小街小巷的路面硬化。实施公共交通优先发展战略，加快公交乘换枢纽建设，继续在快速路和主路上设立公交客运走廊和站点，确立公共交通在城市客运交通体系中的主导地位。

　　(二)创新城市管理机制，提升城市管理水平

　　创新城市管理模式。突出人性化、精细化管理，加强对市容环境、景观亮化、重点区域的综合整治，进一步改善城市面貌和人居环境。建立城市综合管理信息系统，开通“12345”热线，实行常态化、规范化、数字化管理，提高城市管理效率和快捷反应能力。进一步健全市、区、街道(镇)分级管理体制，赋予基层更多的管理权限，充分发挥社区、居委会在城市管理中的积极作用。

　　深化城市环境综合整治。巩固和扩大三年环境综合整治成果，进一步完善相关法规，初步建立长效管理机制。继续推进违法建筑专项整治，遏制城乡新增违法建筑。继续开展项目建设的施工管理，推行文明施工、紧凑施工、规范施工。完成市区农贸市场三年改造方案，启动改造提升工程。以“降事故、保安全、保畅通”为目标，集中力量开展“交通专项整治年”活动，增强市民交通意识，科学系统规划道路交通，提高执法水平，确保交通秩序明显好转。

　　(三)加强环境建设保护，推进“生态海口”建设

　　加大生态环境保护力度。制定《海口生态市建设总体规划》。加强城市环保基础设施建设，切实推进白沙门污水处理厂二期、长流污水处理厂、龙塘污水处理厂建设，进一步完善市区污水截流并网工程，确保污水处理率达到80%以上，切实改善市区地表水体的水质。加强城区垃圾处理站(点)建设，安排1000万元，建设6座垃圾转运站，确保城镇生活垃圾无害化处理率达到90%。全面开展全市污染源普查，加强大气污染、水体污染和噪声污染专项整治。加大植树造林力度，继续建设“百里绿色长廊”乡村公路林、海防林，推进一批街心花园、公共绿地和居民区绿地建设。

　　加强节能降耗和防污减排工作。以节约使用资源和提高资源利用率为核心，建立节约型的生产模式、消费模式和城镇建设模式。对重点行业、重点企业排放实施重点监控，推行清洁生产，推进余热、废水、禽畜养殖业废弃物等综合循环利用。开展生态科学知识和生态法律法规的普及活动，提高市民参与生态文明建设的能力。

　　六、加快发展以改善民生为重点的社会事业

　　今年继续将改善民生作为政府工作的“一号工程”，优先安排资金用于改善民生，实施一批为民办实事事项，确保群众得到更多实惠，努力实现“学有所教、劳有所得、病有所医、老有所养、住有所居”。

　　(一)继续扩大社会就业，努力增加城镇居民收入

　　完善自主创业、自谋职业和灵活就业的鼓励政策，强化创业辅导培训、融资等服务，优化创业环境，支持更多的劳动者成为创业者。加快公共就业服务体系建设，健全城乡一体化的劳动力市场，推进“充分就业社区”创建工作。积极帮扶大中专毕业生等新成长劳动力、“零就业”家庭和其他就业困难人员就业。全年新增就业人员3万人。健全失地农民就业和失业专项登记制度，帮助城镇规划区内失地无业农民就业。

　　(二)坚持教育优先发展，努力办好人民满意的教育

　　安排教育经费1.6亿元，增长19.2%。认真落实“两免一补”政策，不断扩大助学范围。继续完善教育硬件设施建设，着力改善农村中小学办学条件，加快解决学校学位紧张问题。抓好新一轮中小学教师全员培训，加强教育管理和提高师资水平，全面推行素质教育。优化配置城乡教育资源，建立城乡教师交流长效机制，切实推进农村学校规范化建设，推进义务教育均衡发展。加快发展适应产业需求、紧贴市场需要的职业教育，组建旅游、商业和工业等职业教育集团。

　　(三)加强公共卫生体系建设，提高市民的健康水平

　　坚持公共医疗卫生的公益性质，安排医疗卫生经费6405万元，增长153.1%。进一步完善各项卫生应急预案，应对各类突发疫情和公共卫生事件。推进农村卫生院标准化建设，确保村卫生室覆盖率达95%以上。落实社区首诊制和双向转诊制，健全社区卫生服务网络，力争覆盖率达95%。积极探索新型医疗管理体制。

　　(四)进一步完善社会保障体系，保障人民群众的基本生活

　　健全社会保障体系，安排社会保障经费2050万元，增长179.3%。切实推进全民医保工作，扩大城镇居民基本医疗保险覆盖面，确保参保率达95%以上;新型农村合作医疗参合率稳定在95%以上，提高人均参合费补贴标准。建立健全被征地农民基本生活保障制度，切实做到即征即保。完善城乡低保标准自然增长机制，切实做到应保尽保。健全社会救助体系，提高农村五保户集中供养率。采取有效措施保持主要消费品和服务价格的基本稳定，完善低保家庭基本生活消费品价格上涨动态补贴制度和临时救助制度。继续建设廉租房和经济适用房，完成2098套廉租房的建设，切实解决低收入家庭和旧城改造拆迁户的住房问题。

　　(五)推进城乡文明建设，提高文明城市创建水平

　　深入开展创建文明城市工作，加强社会主义核心价值体系建设，完善学校、家庭、社会相结合的未成年人教育网络。积极开展文明行业、文明单位、文明村镇、文明社区创建活动。继续完善公共文化体育基础设施，抓好特色文化街区、镇综合文化站和精神文明活动中心建设，开展丰富多彩的群众性文化体育活动，丰富人民群众的精神文化生活。高标准做好双拥工作，促进军地军民和谐。发挥工会、共青团、妇联等人民团体在创建文明城市中的作用，加强老龄工作，推进妇女儿童和残疾人事业的发展。全面加强人口和计划生育工作，进一步稳定低生育水平，统筹解决人口问题。大力开展爱国卫生运动。继续做好外事侨务、民族宗教、档案史志、地震等工作。

　　(六)完善社会管理功能，提高社会和谐程度

　　围绕构建和谐社会的要求，深入推进平安海口建设。进一步完善社会治安防控体系，认真落实社会治安综合治理各项措施，进一步抓好禁毒工作，严厉打击各类违法犯罪活动。建立和完善人民群众诉求表达机制，进一步落实信访工作责任制，提高矛盾调处工作的针对性和有效性，着力解决人民内部矛盾。完善司法救助机制，加大法律援助力度，促进社会公平正义。大力支持社会团体、行业组织、民办非企业单位和城乡基层自治组织的发展，发挥其协助政府进行社会管理和提供公共服务的作用。建立安全生产、道路交通、食品药品、危险品和消防安全等长效监管机制。完善市应急指挥中心建设，切实保障人民群众生命财产安全。

　　七、切实加强政府自身建设

　　当前，我市正处于发展的上升期，改革发展稳定的各项任务都很重。必须适应新的形势和任务，从人民群众的利益出发，大力加强政府自身建设，不断提高行政效能，努力建设为民务实、廉洁高效的服务型政府。

　　(一) 严格依法行政，切实规范行政行为

　　牢固树立依法行政观念，严格按照法定权限和程序行使权利，依法管理经济社会事务。紧紧围绕改革发展稳定中的重大问题以及关乎民生的热点难点问题，积极配合人大及其常委会做好立法工作，提高政府规章的科学性严谨性。进一步减少和规范行政审批，对已取消的审批项目，切实加强后续监督，坚决杜绝各种变相审批行为。强化行政执法监督，加强执法队伍管理，落实行政执法责任制，提高行政执法水平。

　　(二)强化民主监督，确保权力公正运行

　　全面贯彻执行《中华人民共和国各级人民代表大会常务委员会监督法》，自觉接受人大及其常委会的法律监督和工作监督、政协的民主监督以及社会舆论监督，充分发挥各民主党派、工商联和无党派人士的民主监督作用，进一步提高行政透明度。完善重大决策的程序，对涉及经济社会发展全局和与群众利益密切相关的重大事项，广泛征询意见，严格专家论证，扩大群众参与度，使决策切合实际，符合群众利益。进一步推行政务公开，拓宽新闻发言人、政府网站等政务公开渠道，切实保障人民群众的知情权、参与权、表达权和监督权。

　　(三)推进管理创新，大力提高行政效能

　　围绕把海口打造成全省行政行为规范、服务效能最高的城市，进一步加强政务服务中心建设，形成上下联动的工作机制，提高整体服务水平。进一步理顺部门职能分工，优化工作流程，建立规范高效的执行机制，做到运转更加规范协调、工作更加高效便民。建立健全决策反馈纠偏机制和决策责任追究制度，逐步形成与政府管理创新相适应的激励约束机制和社会评价机制，实行政府内部考核与公众评议、专家评价相结合的绩效考核办法。严格落实行政问责制，认真查处各种不作为、乱作为的行为，进一步优化投资环境。

　　(四)坚持从严治政，进一步加强廉政建设

　　坚持不懈地加强廉政建设，落实“一岗双责”，坚定不移地开展反腐斗争，推进防治腐败体系建设。在坚决惩治腐败的同时，更加注重治本，更加注重预防，更加注重制度建设。加强廉政文化建设，提高领导干部廉洁从政的自觉性。扎实推进政风行风建设，切实纠正损害群众利益的不正之风。加强对实施强区扩权管理体制改革的监督检查，强化对权力的监督制约。认真开展治理商业贿赂专项工作，严肃查办虚假招投标、擅自变更规划设计获取利益等案件。在严肃查处违纪违法行为的同时，鼓励和保护干部的改革创新精神，充分发挥全体公务员的积极性和创造性，提升公务员素质和凝聚力，努力建设一个群众满意的政府。

　　各位代表，全面做好今年的工作，任务艰巨、意义重大。让我们更加紧密地团结在以胡锦涛同志为总书记的党中央周围，在省委、省政府和市委的坚强领导下，团结依靠全市人民，振奋精神，坚定信心，奋发进取，为建设繁荣富裕的海口、环境优良的海口、社会和谐的海口而努力奋斗。
